# IKK*β* Inhibitor IMD-0354 Attenuates Radiation Damage in Whole-body X-Irradiated Mice

**DOI:** 10.1155/2019/5340290

**Published:** 2019-11-27

**Authors:** Kengo Waga, Masaru Yamaguchi, Shuta Miura, Teruki Nishida, Akiko Itai, Reiko Nakanishi, Ikuo Kashiwakura

**Affiliations:** ^1^Department of Radiation Sciences, Hirosaki University Graduate School of Health Sciences, 66-1 Hon-cho, Hirosaki, Aomori 036-8564, Japan; ^2^Institute of Medicinal Molecular Design, Inc., 2-37-6 Hongo, Bunkyo-ku, Tokyo 113-0033, Japan

## Abstract

Nuclear factor-kappa B (NF-*κ*B) transcription factor plays a critical role in regulating radiation-induced inflammatory and immune responses. Intracellular reactive oxygen species generation induces the activation of NF-*κ*B via the inhibitor of *κ*B (I*κ*B) kinase (IKK) complex signaling. Previous studies have reported that the inhibition of IKK-driven NF-*κ*B activation offers a therapeutic strategy for managing inflammatory disorders and various cancers, but it has additionally been reported that treatment targeting NF-*κ*B also shows a radioprotective effect. IMD-0354 is an IKK*β* inhibitor that blocks I*κ*B*α* phosphorylation in the NF-*κ*B pathway. This compound is known to exert anti-inflammatory and antitumor effects, but its radioprotective effects are unclear. Therefore, in the present study, we examined whether or not IMD-0354 has a mitigative effect on radiation-induced damages in mice. IMD-0354 was dissolved in soybean oil and subcutaneously administered to C57BL/6J Jcl mice for 3 consecutive days after 7 Gy of whole-body X-irradiation. The survival rate on day 30 and the NF-*κ*B p65 and I*κ*B*α* in bone marrow and spleen cells based on flow cytometry were assessed. IMD-0354 administration significantly suppressed the lethality induced by whole-body X-irradiation, and the survival rate increased by 83%. The NF-*κ*B p65 and I*κ*B*α* in bone marrow and spleen cells were significantly lower in IMD-0354-treated mice than in irradiated mice, suggesting that the IKK*β* inhibitor IMD-0354 exerts a radiomitigative effect by suppressing the NF-*κ*B.

## 1. Introduction

Nuclear factor-kappa B (NF-*κ*B) is involved in many physiological phenomena such as immune reaction and cell death, as well as in the regulation of cell proliferation and apoptosis [1]. Activation of the inhibitor of *κ*B (I*κ*B) kinase (IKK) complex signaling occurs in response to extracellular stresses such as radiation, inflammation, and reactive oxygen species [2]. Activation of IKK*β* which is a part of the IKK complex mainly occurs in the canonical NF-*κ*B pathway [3]. NF-*κ*B is usually present in the cytoplasm in association with I*κ*B*α* that suppresses the nuclear translocation of NF-*κ*B, but activation of IKK*β* causes phosphorylation of I*κ*B*α* by serine residues [4, 5]. Phosphorylated I*κ*B*α* is then ubiquitinated and becomes the target of degradation by the 26S proteasome, and NF-*κ*B detached from I*κ*B*α* migrates into the nucleus and binds to DNA, resulting in the gene expression of inflammatory proteins, antiapoptotic proteins, or cell-adhesion molecules [5–8]. Since NF-*κ*B is constitutively activated in many cancer cells, several studies have evaluated substances targeting NF-*κ*B as anticancer agents [9, 10].

IMD-0354, a specific IKK*β* inhibitor, is a low molecular weight compound selected based on inhibition of NF-*κ*B activation in cultured cells under the stimulation of tumor necrosis factor-*α* (TNF-*α*) [11]. IMD-0354 has been studied as an anticancer agent and is reported to be effective in the treatment of pulmonary fibrosis, myocardial ischemia, atopic dermatitis, adult T cell leukemia, bronchial asthma, and autoimmune myocarditis, and it has been confirmed that it decreases proteins such as TNF-*α* and interleukin 1*β* (IL-1*β*) [12–17]. Inhibition of NF-*κ*B blocks the production of inflammatory cytokines and vascular endothelial growth factors; suppresses angiogenesis, metastasis, and invasion in cancer; and induces cell death [18–21]. Since NF-*κ*B is inactivated in normal cells, IMD-0354, a highly specific kinase inhibitor, has drawn attention recently as a new anticancer drug showing no toxicity for normal cells [4, 22]. Actually, it has been confirmed that IMD-0354 is not toxic in animal experiments [12, 13]; the prodrug has been confirmed to be highly safe in the P-1 test in healthy persons and in the P-2a study, which examines insulin resistance in type 2 diabetes. In normal cells, NF-*κ*B is activated in response to radiation and induces the production of inflammatory cytokines, thereby damaging the cells [23]. Recently, compound targeting NF-*κ*B was reported to exert a radioprotective effect [23, 24]. Therefore, IMD-0354 can be expected to show not only inflammation-suppressing and antitumor effects on cancer cells but also a radiation-mitigating effect in irradiated individuals. However, the radiation protective effect of IMD-0354 has not yet been described.

In the present study, to assess the radiomitigative potential of the IKK*β* inhibitor IMD-0354, the survival rate of mice at day 30 and the NF-*κ*B and I*κ*B*α* in bone marrow and spleen cells using flow cytometry were assessed.

## 2. Materials and Methods

### 2.1. Animal Experiments

Seven-week-old female C57BL/6J Jcl inbred mice were purchased from Japan Clea Corporation (Kanagawa, Japan). Mice were acclimatized at an animal husbandry facility at Hirosaki University Graduate School of Health Sciences under a light/dark cycle of 12 h, with food and water available *ad libitum*, and had their characteristics, such as body weight, measured over time. The breeding and experimental protocols for the mice were approved by the animal experiment committee of Hirosaki University and carried out while strictly observing the provisions concerning animal experiments at Hirosaki University prescribed by the committee. In the present study, select criteria were applied prior to sacrifice: a more than 20% loss of body weight and respiratory distress. The number of mice used for each experiment is indicated in the figure legends.

### 2.2. X-Ray Total-Body Irradiation (TBI) in Mice

Eight-week-old mice were subjected to whole-body irradiation of 5 Gy or 7 Gy of X-irradiation (150 kVp, 20 mA, 0.5 mm aluminum and 0.3 mm copper filters) at a dose rate of 1.0 Gy/min using an X-irradiation generator (MBR-1520R; Hitachi Medical Co., Tokyo, Japan).

### 2.3. Drug Administration

The IKK*β* inhibitor IMD-0354 (Lot. A-01 R1-JF1) was provided by the Institute of Medicinal Molecular Design (Tokyo, Japan). The weighed IMD-0354 powder was added to soybean oil (Lot. WDR2269; Wako Pure Chemical Co., Osaka, Japan) to prepare a suspension solution. After preparation, it was kept under refrigerated light protection, and at the time of administration, it was warmed to 37°C in a water bath and administered after resuspension. Within 2 h after TBI, IMD-0354 was subcutaneously administered once daily for 3 days at a dosage of 5 mg/kg of body weight/day to X-irradiated mice. X-irradiated mice with soybean oil treatment were used as controls.

### 2.4. Collection of Bone Marrow Cells and Spleen Cells

For X-irradiated mice, both femurs were collected from each mouse after treatment with isoflurane-containing escafine-containing inhalation anesthetic solution (Mylan Pharmaceutical Co., Ltd., Osaka, Japan) on days 4, 8, and 18 after irradiation. Flashing with 0.5% bovine serum albumin (BSA)/ethylenediamine-N,N,N′,N′-tetraacetic acid (EDTA)/calcium-magnesium-free phosphate-buffered saline (PBS (-)) (BSA-EDTA-PBS) was performed to recover bone marrow cells. At the same time, spleens were collected from each mouse and sown on a mesh filter and spleen cells were collected with calcium-magnesium-contain Hanks' Balanced Salt Solution (HBSS (+)) (HBSS). The spleen weight was also measured at the time of collection. The collected cells were centrifuged at 400 g, 4°C for 10 minutes, and the sediment was resuspended in 0.5% BSA-EDTA-PBS. Hemolytic Gey salt solution was added, and hemolysis treatment was performed on ice for 5 minutes. After treatment, centrifugation was carried out at 2000 rpm for 3 minutes, the sediment was resuspended in 0.5% BSA-EDTA-PBS, and the number of viable cells was calculated by the trypan blue dye exclusion method.

### 2.5. An Analysis of NF-*κ*B p65 and I*κ*B*α*

A total of 5.0 × 10^5^ viable cells were dispensed, and fixation was performed using eBioscience™ Foxp3/Transcription Factor Staining Buffer Set (Thermo Fisher Scientific, Tokyo, Japan). After washing and centrifugation at 400 g, 4°C, 5 min with Permeabilization Buffer 10x (Thermo Fisher Scientific), I*κ*B*α* Monoclonal Antibody (T.937.7) (Thermo Fisher Scientific) and NF-*κ*B p65 Polyclonal Antibody (Thermo Fisher Scientific) were added (30 min, at room temperature with a light shield). After washing and centrifugation at 400 g, 4°C, 5 min with Permeabilization Buffer 10x, Goat anti-Mouse IgG (H+L) Cross-Adsorbed Secondary Antibody, Alexa Fluor 546 (Thermo Fisher Scientific), and Goat anti-Rabbit IgG (H+L) Cross-Adsorbed Secondary Antibody, Alexa Fluor 488 (Thermo Fisher Scientific), were added (1 h, at room temperature with a light shield). Permeabilization Buffer 10x was then added, and the mixture was washed and centrifuged twice at 400 g, 4°C for 5 min, and analyzed with a flow cytometer. The cells were then adhered to microscope glass slides (Matsunami Glass Ind., Osaka, Japan) using a StatSpin® CytoFuge 2 (Iris Sample Processing, Inc., Westwood, MA, USA) and mounted using a Vectashield® Mounting Medium with 4′,6-diamidino-2-phenylindole (DAPI) (Vector Laboratories, Inc., Burlingame, CA, USA). Images of cell nuclei, NF-*κ*B, and I*κ*B*α* were obtained using an LSM 710 laser scanning microscope (Carl Zeiss Microscopy Co., Ltd., Tokyo, Japan).

### 2.6. Profiling Hematopoietic Stem/Progenitor Cells in Bone Marrow and Spleen

Hematopoietic differentiation profiles of bone marrow cells and splenic cells were analyzed using FACSAria (Becton Dickinson, Franklin Lakes, NJ, USA). Each from bone marrow and splenic single cell suspension, 2.5 × 10^5^ cells were divided into a new tube and stained with antimurine CD117 (c-kit), Ly6A/E (Sca-1), and CD34 antibodies conjugated with different types of fluorophores and phycoerythrin- (PE-) conjugated antibody cocktail involving antimurine CD11b, CD45R/B220, CD8a, Ly6G/Ly6C (Gr-1), and TER119 antibodies. Then, the fluorescence-labelled cells were staining with 7AAD (Becton Dickinson) and analyzed with flow cytometry. We gated 7AAD^–^ viable cell population and counted the numbers of Lin^–^ c-kit^+^ Sca-1^+^ CD34^–^(population enriched for hematopoietic stem and progenitor cells), Lin^–^ c-kit^+^ Sca-1^+^ CD34^+^ (multipotent progenitor), Lin^–^ c-kit^+^ Sca-1^–^ CD34^+^ (common myeloid-erythroid progenitor), and Lin^–^ c-kit^–^ Sca-1^+^ CD34^+^ (common lymphoid progenitor) cell populations. Above monoclonal antibodies (mAbs) were purchased from the following suppliers: Becton Dickinson (allophycocyanin- (APC-) cyanin-7-forochrome- (APC-Cy7-) conjugated Sca-1 mAb, lineage markers containing PE-conjugated CD11b, CD45R/B220, CD8a, Ly6G/Ly6C, and TER119 mAbs), BioLegend (San Diego, CA, USA) (PE-Cy7-conjugated c-kit mAb), and Thermo Fisher Science (Alexa Fluor 700 conjugated CD34 mAb (RAM34)).

### 2.7. Statistical Analyses

Significant differences were assessed using Student's *t*-test, and the significance level was set at ≤5%. Statistical processing was performed using the Statcel 3 software program (OMS Publishing, Saitama, Japan). To assess significance differences in the survival rate, the log-rank test was performed and the significance level was set at ≤5%. Statistical processing was performed using the Excel 2010 software program (Microsoft, Redmond, Washington, USA).

## 3. Results

### 3.1. Effects of IMD-0354 on the Survival Rate of X-Irradiated Mice

**Figure 1:**
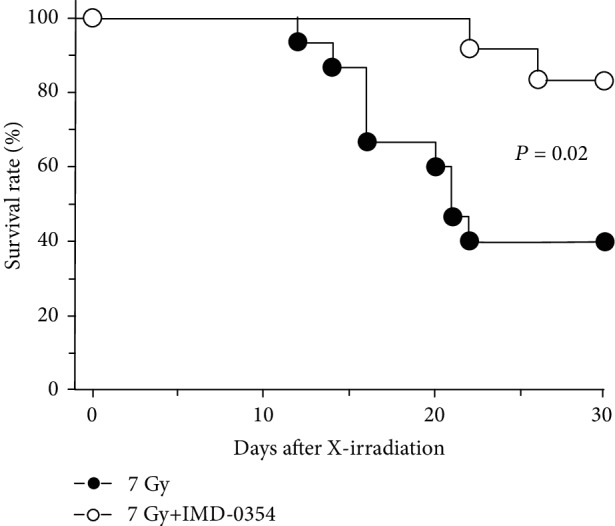
The survival rate of X-irradiated mice administered IMD-0354. The survival curves at 30 days after 7 Gy X-irradiation in the IMD-0354-treated and untreated groups of mice are shown. IMD-0354 was added to soybean oil and subcutaneously administered once daily for 3 consecutive days at 5 mg/kg/day to X-irradiated mice (“7 Gy+IMD-0354” group, *n* = 12), and soybean oil was administered alone to the irradiated control group (“7 Gy” group, *n* = 15). Survival data were analyzed using Kaplan-Meier survival curves.

IMD-0354 was subcutaneously administered to whole-body X-irradiated mice for 3 days, and the survival rate was evaluated over 30 days (Figure 1). In the irradiated mice without IMD-0354 administration, individual death was observed from day 12 after irradiation and the survival rate on day 30 was 40%. The administration of IMD-0354 to X-irradiated mice significantly improved the survival rate on day 30 to 83%, showing that IMD-0354 possesses a radiation-mitigating effect for X-irradiated individuals.

### 3.2. Effects of IMD-0354 on the Spleen and Bone Marrow of X-Irradiated Mice

**Figure 2:**
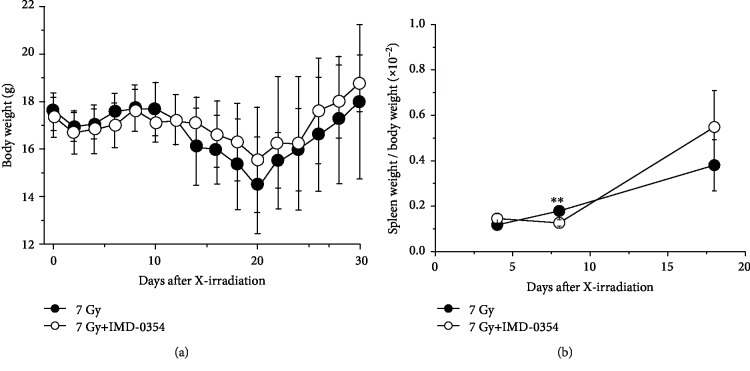
The body weight and ratio of spleen weight to body weight in X-irradiated mice administered IMD-0354. Changes in the body weight (a) and the ratio of spleen weight to body weight (b) collected from 7 Gy X-irradiated mice in the IMD-0354-treated and untreated groups were shown. Spleens were collected from mice on days 4, 8, and 18 after X-irradiation, and the weight was measured, and the value divided by the mouse body weight. Data represent the means ± SD ((a): 7 Gy group, *n* = 15, 7 Gy+IMD-0354, *n* = 12. (b): *n* = 3‐6). ^∗∗^*P* < 0.01: 7 Gy irradiation group vs. 7 Gy irradiation+IMD-0354 group.

To evaluate the effect of IMD-0354 on the body weight and spleen of X-irradiated mice, body weight and ratios of spleen weight to body weight of mice were estimated (Figure 2). In both groups, body weight decreased greatly on day 20 after irradiation and then increased (Figure 2(a)). In X-irradiated mice without IMD-0354 administration, this ratio increased from days 4 to 18 after irradiation (Figure 2(b)). In mice with IMD-0354 administration, the spleen weight ratio was decreased on day 8 (*P* < 0.01), but no significant difference was observed on day 18.

**Figure 3:**
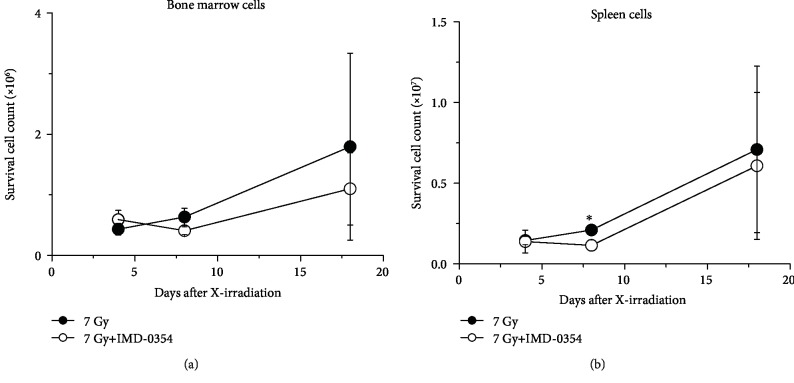
An analysis of the viable cell count in X-irradiated mice administered IMD-0354. Time-dependent changes in the bone marrow viable cell count (a) and the spleen viable cell count (b) in 7 Gy X-irradiated mice in the IMD-0354-treated and untreated groups are shown. The spleen and bone marrow were collected from mice on day 4, 8, and 18 after X-irradiation, and the viable cell count was calculated by the trypan blue dye exclusion method. The 0 Gy irradiated group was indicated by the dotted line. Data represent the means ± SD (*n* = 3‐6). ^∗^*P* < 0.05: 7 Gy irradiation group vs. 7 Gy irradiation+IMD-0354 group.

In both groups, the number of viable cells observed in bone marrow and spleen was counted by the trypan blue dye exclusion method (Figure 3). There was no significant difference between the two groups in the number of bone marrow cells (Figure 3(a)). However, a significant difference was observed in the number of spleen cells on day 8, but both cell numbers increased similarly during the observation period (Figure 3(b)). In addition, as a result of preliminary experiments regarding hematopoietic stem cells in bone marrow cells and spleen cells obtained from 5 Gy irradiated mice with a flow cytometer, IMD-0354 had no effect on nonirradiated mice on day 7. On the other hand, administration of IMD-0354 to irradiated mice significantly improved the number of hematopoietic stem/progenitor cells on day 30 compared to irradiated mice. In particular, multipotent progenitors were significantly improved in bone marrow cells and common lymphoid progenitors were significantly improved in spleen cells (Table 1).

### 3.3. Effects of IMD-0354 on the NF-*κ*B p65 and I*κ*B*α* in Bone Marrow and Spleen Cells

**Figure 4:**
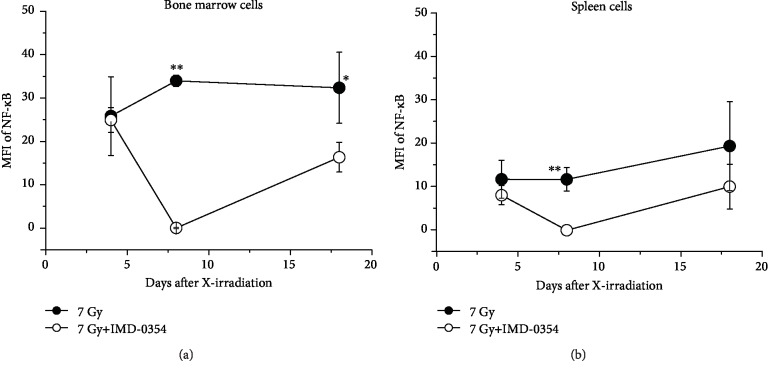
A NF-*κ*B analysis in X-irradiated mice administered IMD-0354. The MFI of NF-*κ*B in the bone marrow (a) and the spleen (b) in 7 Gy X-irradiated mice in the IMD-0354-treated and untreated groups is shown. NF-*κ*B was labeled with NF-*κ*B p65 Polyclonal Antibody and Goat anti-Rabbit IgG (H+L) Cross-Adsorbed Secondary Antibody, Alexa Fluor 488 and subjected to flow cytometry. Data represent the means ± SD (*n* = 3‐6). ^∗^*P* < 0.05, ^∗∗^*P* < 0.01: 7 Gy irradiation group vs. 7 Gy irradiation+IMD-0354 group.

**Figure 5:**
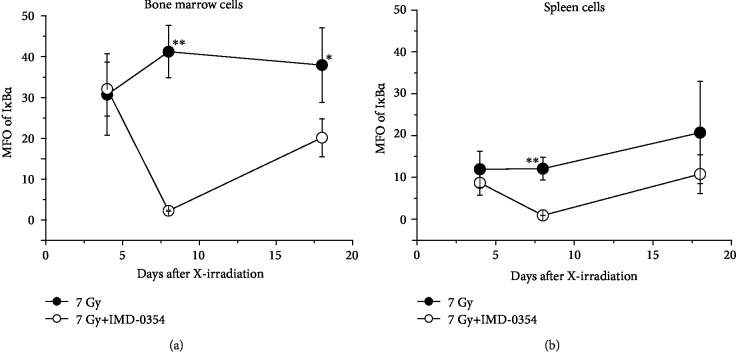
An I*κ*B*α* analysis in X-irradiated mice administered IMD-0354. The MFI of I*κ*B*α* in the bone marrow (a) and the spleen (b) in 7 Gy X-irradiated mice in the IMD-0354-treated and untreated groups is shown. I*κ*B*α* was labeled by I*κ*B*α* Monoclonal Antibody (T.937.7) and Goat anti-Mouse IgG (H+L) Cross-Adsorbed Secondary Antibody, Alexa Fluor 546 and subjected to flow cytometry. Data represent the means ± SD (*n* = 3‐6). ^∗^*P* < 0.05, ^∗∗^*P* < 0.01: 7 Gy irradiation group vs. 7 Gy irradiation+IMD-0354 group.

In order to estimate the IKK*β* inhibitory action of IMD-0354 in X-irradiated mice, the mean fluorescence intensity (MFI) of NF-*κ*B in bone marrow and spleen cell was evaluated (Figure 4). Regarding bone marrow cells, IMD-0354 administration resulted in a sharp decline in the NF-*κ*B on day 8, although its level had almost recovered by day 18 (Figure 4(a)). In contrast, the NF-*κ*B increased in X-irradiated mice without IMD-0354 administration, and a significant difference was observed on days 8 and 18 in cells from mice treated with IMD-0354 (Figure 4(a)). Similar trends were observed in spleen cells, but a significant difference was observed only on day 8 (Figure 4(b)). In addition, the I*κ*B*α* was also evaluated in bone marrow and spleen cells (Figure 5). In both cells, the I*κ*B*α* showed a variation similar to the changes in the NF-*κ*B, and a significant difference was observed in bone marrow cells on days 8 and 18 (Figure 5(a)) and in spleen cells on day 8 (Figure 5(b)).

**Figure 6:**
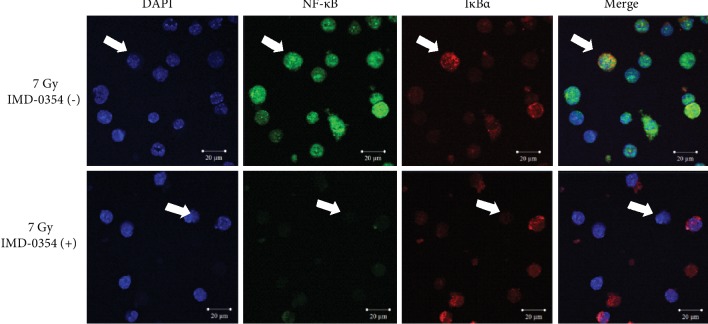
An analysis of the bone marrow cell NF-*κ*B and I*κ*B*α* in X-irradiated mice administered IMD-0354. Representative images of NF-*κ*B and I*κ*B*α* in bone marrow cells collected from 7 Gy X-irradiated mice on day 8 after irradiation are shown. The bone marrow cells in the femurs were collected from irradiated mice with or without IMD-0354 treatment, and the NF-*κ*B and I*κ*B*α* were evaluated by an LSM 710 laser scanning microscope. Blue: DAPI (nucleus), green: NF-*κ*B, and red: I*κ*B*α*. The scale bar is 20 *μ*m. White arrows indicate representative cells.

**Figure 7:**
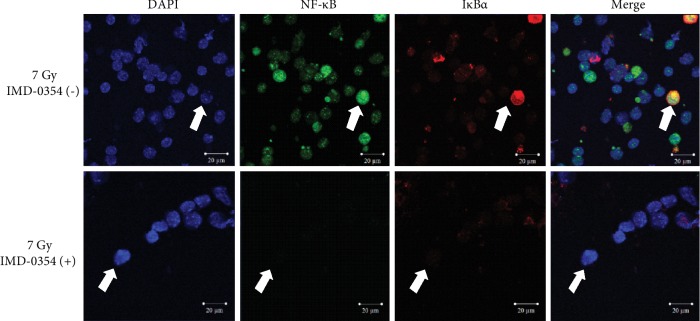
An analysis of the spleen cell NF-*κ*B and I*κ*B*α* in X-irradiated mice administered IMD-0354. Representative images of NF-*κ*B and I*κ*B*α* in the spleen cell collected from 7 Gy X-irradiated mice on day 8 after irradiation are shown. The spleen cells were collected from irradiated mice with or without IMD-0354 treatment, and the NF-*κ*B and I*κ*B*α* were evaluated by an LSM 710 laser scanning microscope. Blue: DAPI (nucleus), green: NF-*κ*B, and red: I*κ*B*α*. The scale bar is 20 *μ*m. White arrows indicate representative cells.

Regarding the visual assessment of the nuclear translocation of NF-*κ*B and degradation of I*κ*B*α* in bone marrow and spleen cells of X-irradiated mice, representative images of each obtained by a confocal laser scanning microscope are shown in Figures 6 and 7. In the bone marrow cells of 7 Gy irradiated mice (Figure 6), both NF-*κ*B (green) and I*κ*B*α* (red) were observed in nuclei stained blue with DAPI (as indicated by white arrows). In contrast, the NF-*κ*B and I*κ*B*α* were decreased with the administration of IMD-0354. Similarly, the NF-*κ*B and I*κ*B*α* were also clear in the nuclei of spleen cells derived from 7 Gy irradiated mice (Figure 7), while IMD-0354 reduced the NF-*κ*B and I*κ*B*α*.

## 4. Discussion

In the present study, the radiomitigative potential of the IKK*β* inhibitor IMD-0354 was examined in 7 Gy X-irradiated mice. The survival rate of X-irradiated control mice was 40% on day 30 after irradiation, while the administration of IMD-0354 significantly increased the survival rate to 83% in treated mice, showing the radiomitigative potential of IMD-0354 (Figure 1). In contrast, no remarkable differences were observed in the spleen weight ratio or in the number of bone marrow or spleen cells in X-irradiated mice regardless of IMD-0354 administration, except on day 8 (Figures 2 and 3). Since the radiation dose of 7 Gy used in the present study causes individual death due to a decrease in hematopoietic stem/progenitor cells [25], the maintenance of hematopoietic function is extremely important in the survival of radiation exposure individuals. Although we did not confirm the detailed mechanism underlying the effects of IMD-0354 on DNA damage and hematopoietic function in bone marrow and spleen cells, the present results suggest that IMD-0354 may have less influence on the hematopoiesis directly of X-irradiated mice.

IKK is a complex of three kinase subunits: IKK*α*, IKK*β*, and IKK*γ*. NF-*κ*B is activated by the IKK*β* subunit, while the other two play a little or no role in the canonical pathway [3, 26]. Therefore, previous studies have focused on IKK*β* [27–29]. I*κ*B*α* is phosphorylated and decomposed with the activation of IKK*β*, and NF-*κ*B dimer released from the cytoplasmic NF-*κ*B-I*κ*B complex causes transcription in the nucleus [5, 7, 8]. In various diseases, NF-*κ*B dysregulation in the canonical pathway has been confirmed, and the dysregulation of NF-*κ*B is also activated by radiation exposure [9, 10, 12, 24]. In the present study, the administration of IMD-0354 to X-irradiated mice significantly suppressed the NF-*κ*B in bone marrow cells on days 8 and 18 and in spleen cells on day 8 after X-irradiation (Figures 4, 6, and 7). Similarly, IMD-0354 significantly inhibited the I*κ*B*α* in bone marrow cells on days 8 and 18 and spleen cells on day 8 after irradiation (Figures 5–7). Although NF-*κ*B is inactivated in normal cells, NF-*κ*B is constitutively activated in many cancer cells, promoting proliferation, antiapoptosis, and angiogenesis in addition to transcription [4, 18, 19]. Since the misregulation of NF-*κ*B is associated with various diseases, including inflammation and cancers [4, 21, 22], the IKK*β* inhibitor IMD-0354 has been investigated as an anticancer agent, and by suppressing NF-*κ*B, a significant reduction in tumor size in breast cancer xenograft mice and a significant decrease in tumor cells and tumor size in pancreatic cancer xenograft mice have been reported [4, 22]. Wang et al. reported that the administration of ursolic acid contained in herbs to 6.4 Gy irradiated mice which is the radiation dose at which ≥20% of the mice die, significantly suppressed NF-*κ*B and improved the survival rate to 100% [23]. In addition, Kalita et al. reported that the administration of G-003M, which is an anti-inflammatory agent created by combining the anticancer agent podophyllotoxin and anti-inflammatory substance rutin at a ratio of 1 : 2 to 9 Gy irradiated mice significantly suppressed the NF-*κ*B and improved the survival rate from 0% to 89% [24]. The present results showed that NF-*κ*B is increased by X-irradiation, and the suppression of NF-*κ*B was shown to be effective in reducing radiation damages, thus suggesting that the IKK inhibitor IMD-0354 may be an effective means of reducing radiation damage.

NF-*κ*B promotes the expression of proinflammatory cytokines such as IL-1*β* and TNF-*α* [30] and is considered a major factor of the positive feedback of inflammation. It has been confirmed that inflammatory cytokines, such as IL-1*β*, TNF-*α*, and IL-6 in macrophages and TNF-*α* in hepatocytes, were suppressed by IMD-0354 [31–33]. The present findings suggest that suppression of inflammatory positive feedback may be involved in ameliorating the reduction in the survival rate by high-dose irradiation.

Since IMD-0354 was developed as a selective inhibitor of IKK*β* and its specificity of effect is high, it is expected to exert a damage-mitigating effect against oxidative stress, such as that caused by radiation. However, further details regarding the effects of IMD-0354 will be required for its application to treating radiation exposure.

## 5. Conclusions

The IKK*β* inhibitor IMD-0354 significantly improved the survival rate of mice receiving 7 Gy X-irradiation from 40% of X-irradiated mice without IMD-0354 to 83% on day 30. The NF-*κ*B and I*κ*B*α* detected in bone marrow and spleen cells of IMD-0354-treated mice significantly decreased compared without IMD-0354, suggesting that the IKK*β* inhibitor IMD-0354 exhibits a radiation-mitigating effect by suppressing the NF-*κ*B.

## Figures and Tables

**Table 1:** Profiling hematopoietic stem/progenitor cells in bone marrow and splenic cells.

Bone marrow cells	Days	0 Gy	5 Gy	5 Gy+IMD-0354
		Cell counts (×10^6^ cells)		
Population enriched with hematopoietic stem/progenitor cells	Day 7	0.10 ± 0.03	0.01 ± 0.00	0.01 ± 0.00
	Day 30	ND	0.11 ± 0.01	0.17 ± 0.01^***b***^
Multipotent progenitor	Day 7	0.11 ± 0.04	0.01 ± 0.00	0.01 ± 0.01
	Day 30	ND	0.11 ± 0.01	0.17 ± 0.01^***b***^
Common myeloid-erythroid progenitor	Day 7	0.17 ± 0.04	0.01 ± 0.00	0.01 ± 0.01
	Day 30	ND	0.17 ± 0.06	0.15 ± 0.06
Common lymphoid progenitor	Day 7	0.03 ± 0.00	0.01 ± 0.00	0.01 ± 0.00
	Day 30	ND	0.05 ± 0.01	0.04 ± 0.02

Splenic cells	Days	0 Gy	5 Gy	5 Gy+IMD-0354
Population enriched with hematopoietic stem/progenitor cells	Day 7	0.24 ± 0.01	0.01 ± 0.00	0.01 ± 0.01
	Day 30	ND	0.13 ± 0.05	0.33 ± 0.08^***a***^
Multipotent progenitor	Day 7	0.24 ± 0.02	0.01 ± 0.00	0.01 ± 0.01
	Day 30	ND	0.18 ± 0.13	0.32 ± 0.08
Common myeloid-erythroid progenitor	Day 7	0.01 ± 0.02	0.00 ± 0.00	0.00 ± 0.00
	Day 30	ND	0.05 ± 0.02	0.06 ± 0.01
Common lymphoid progenitor	Day 7	0.26 ± 0.00	0.04 ± 0.01	0.03 ± 0.01
	Day 30	ND	0.38 ± 0.08	0.74 ± 0.06^*b*^

Each cell population was classified according to the following surface antigens. Population enriched with hematopoietic stem/progenitor cells: Lin^–^ c-kit^+^ Sca-1^+^ CD34^–^; multipotent progenitor: Lin^–^ c-kit^+^ Sca-1^+^ CD34^+^; common myeloid-erythroid progenitor: Lin^–^ c-kit^+^ Sca-1^–^ CD34^+^; common lymphoid progenitor: Lin^–^ c-kit^–^ Sca-1^+^ CD34^+^. The data are expressed as the means ± SD, and statistical significance was determined by a comparison of each group (*^a^P* < 0.05*vs*. 5 Gy cohort; *^b^P* < 0.01*vs*. 5 Gy cohort). ND: no data.

## Data Availability

All figure data used to support the findings of this study are available from the corresponding author upon request.
